# The genome and diet of a 35,000‐year‐old *Canis lupus* specimen from the Paleolithic painted cave, Chauvet‐Pont d'Arc, France

**DOI:** 10.1002/ece3.9238

**Published:** 2022-08-23

**Authors:** Jean‐Marc Elalouf, Pauline Palacio, Céline Bon, Véronique Berthonaud, Frédéric Maksud, Thomas W. Stafford, Christophe Hitte

**Affiliations:** ^1^ Institute for Integrative Biology of the Cell (I2BC) Institut des Sciences du vivant Frédéric Joliot, CNRS UMR 9198, CEA Saclay Gif‐sur‐Yvette cedex France; ^2^ Eco‐anthropologie, Muséum National d'Histoire Naturelle, CNRS UMR 7206 Université de Paris Paris France; ^3^ Service Régional de l'Archéologie Toulouse cedex 6 France; ^4^ Stafford Research Albuquerque New Mexico USA; ^5^ Univ Rennes, CNRS, IGDR‐UMR 6290 Rennes France

**Keywords:** ancient DNA, canid, cave bear, Paleogenomics

## Abstract

The Chauvet‐Pont‐d'Arc Cave (Ardèche, France) contains some of the oldest Paleolithic paintings recorded to date, as well as thousands of bones of the extinct cave bear, and some remains and footprints of other animals. As part of the interdisciplinary research project devoted to this reference cave site, we analyzed a coprolite collected within the deep cave. AMS radiocarbon dating of bone fragments from the coprolite yielded an age of 30,450 ± 550 RC yr. BP (AAR‐19656; 36,150–34,000 cal BP), similar to ages assigned to Paleolithic artwork and cave bear remains from the same cave sector. Using high‐throughput shotgun DNA sequencing, we demonstrated a high abundance of canid DNA and lesser amounts of DNA from the extinct cave bear. We interpret the sample as feces from a canid that had consumed cave bear tissue. The high amount of canid DNA allowed us to reconstruct a complete canid mitochondrial genome sequence (average coverage: 83×) belonging to a deeply divergent clade of extinct mitochondrial wolf lineages that are most closely related to coeval (~35 ka) Belgian wolves. Analysis of the nuclear genome yielded a similar coverage for the X chromosome (2.4×) and the autosomes (range: 2.3–3.2×), indicating that the Chauvet canid was a female. Comparing the relationship of the nuclear genome of this specimen with that of a variety of canids, we found it more closely related to gray wolves' genomes than to other wild canid or dog genomes, especially wolf genomes from Europe and the Middle East. We conclude that the coprolite is feces from an animal within an extinct wolf lineage. The consumption of cave bear by this wolf likely explains its intrusion into the dark cave sectors and sheds new light on the paleoecology of a major cave site.

## Graphical abstract

We analyzed a coprolite (fossil feces) from the Paleolithic painted cave of Chauvet‐Pont d'Arc (Ardèche, France). AMS radiocarbon dated yielded an age of 30,450 ± 550 radiocarbon yr. BP (36,150–34,000 cal BP), similar to ages assigned to Paleolithic artwork from the same cave sector. Using high‐throughput ancient DNA sequencing, we demonstrate that the coprolite corresponds to feces produced by a female gray wolf that had consumed cave bear tissue; the Chauvet wolf specimen is more related to Eurasian gray wolves than to domestic dogs, especially ancient Western Eurasian wolves that delineate an extinct lineage.

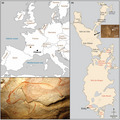

## 1 INTRODUCTION

**FIGURE 1:**
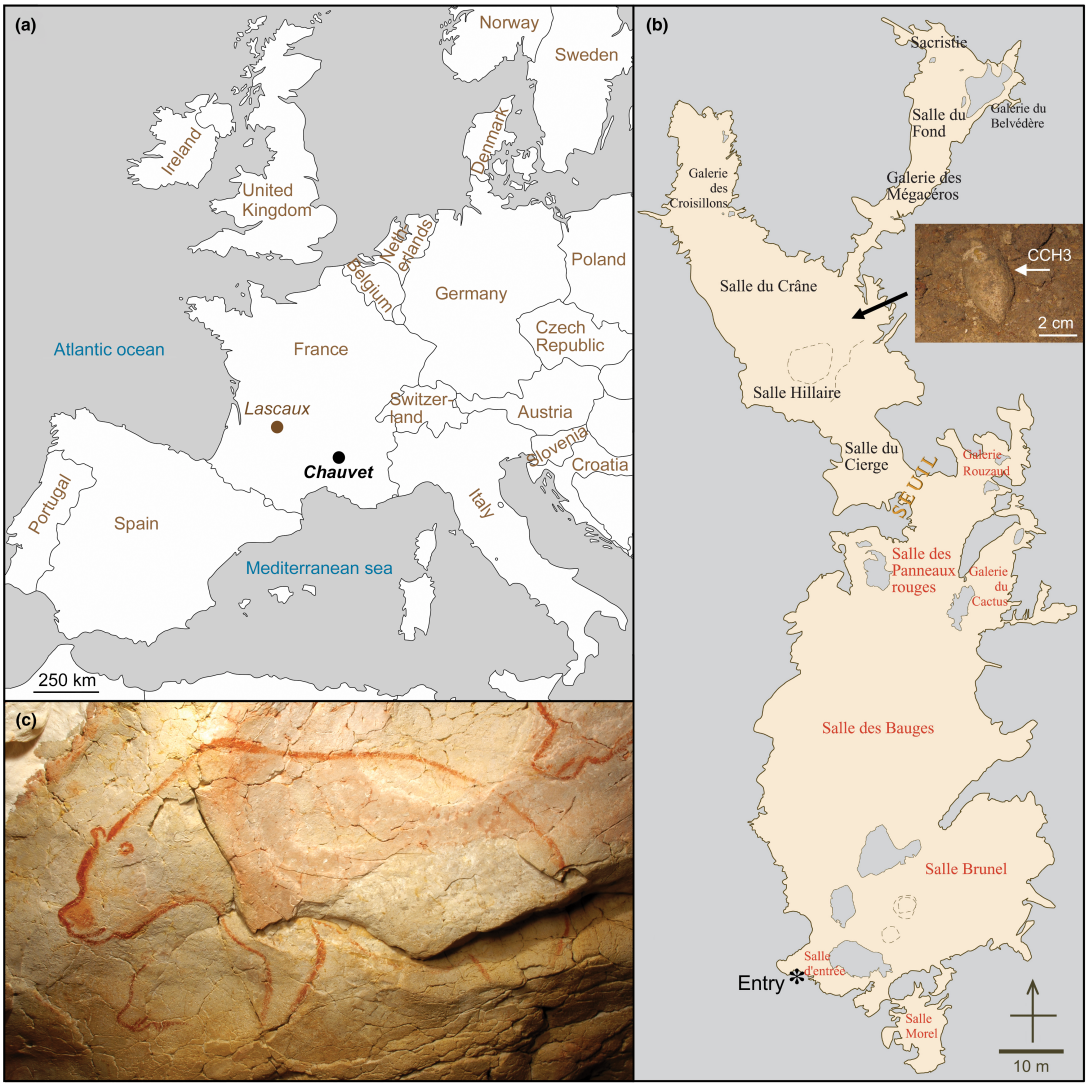
Cave location and interior. (a) Geographic location of the Chauvet‐Pont d'Arc Cave. For information, the location of the Lascaux Cave is also indicated. (b) Topography of the cave showing the position of the coprolite sample in the Hillaire chamber. Red and black characters refer to the color of rock art pictures in the entry and deep cave sectors, respectively. (c) Painting of extinct cave bear in Salle Brunel; photograph by Valérie Feruglio, Ministère de la Culture.

Cave sites are major resources for investigating past and present environments, species adaptation, and human evolution. These deposits span time scales ranging from millions of years (e.g., bedrock geology and cave formation), to tens of thousands of years (human evolution, paleontology, archeology), and finally to the present (climate recording for conservation policy). Some caves are famous for their abundant mineralization, animal remains, or traces of humans, the latter including footprints, hearths, lithic artifacts, or rock art. Chauvet Cave (Figure 1), discovered in 1994, is unique for the simultaneous presence and remarkable preservation of all such items, which supported the cave's assignment to the World Heritage List of United Nations Educational Scientific and Cultural Organization (UNESCO). The cave contains the best‐preserved Aurignacian painted rock art, dated to 33,500–37,000 cal BP (Quiles et al., 2016). Sealing of the cave entrance by rock fall 21,000 years ago (Sadier et al., 2012) and the strict conservation strategy implemented since its 1994 discovery (Delannoy & Geneste, 2020) have enabled the conservation of pristine wall‐rock paintings and the cave's wealth of archeological and animal remains.

Fossil animal bones are abundant in the Chauvet Cave, with over 4000 fossil specimens having been recorded throughout the cave (Delannoy & Geneste, 2020; Fosse & Philippe, 2005). The majority of the fossils (number of remains [NR] > 3700) are skeletal remains of the extinct cave bear (*Ursus spelaeus*). Other Pleistocene remains, in decreasing abundance include, *Capra ibex* (NR = 67), *Vulpes vulpes* (NR = 32), *Canis lupus* (bones, NR = 14; coprolites, NR = 10), and *Cuon alpinus* (NR = 6; Delannoy & Geneste, 2020; Fosse et al., 2020; Fosse & Philippe, 2005; Garcia, 2005). We previously demonstrated the good preservation of ancient DNA in cave bear bones, which provided the complete mitochondrial genome sequence of a 36,000‐year‐old individual (Bon et al., 2008). *Canis lupus* bones from the cave's entry and its deep sectors have been radiocarbon dated 28,930 ± 250 BP (GrA33573) (33,700–32,400 cal BP) and 31,260 ± 190 BP (GrA32592) (35,600–34,800 cal BP), respectively (Bon et al., 2011; Quiles et al., 2016); however, ancient DNA analysis of these fossils was unsuccessful. Because coprolites contain high amounts of ancient DNA (Bon et al., 2012), we analyzed what resembled a *Canis lupus* coprolite to obtain additional ancient DNA data for Chauvet Cave animals.

The gray wolf (*Canis lupus*) is the direct ancestor of domestic dogs. A conservative estimate suggests that dog domestication was initiated about 25,000 years ago (as reviewed in Freedman & Wayne, 2017), and Perri et al. (2021) put forward the hypothesis that it took place in Siberia rather than in Europe or the Middle East. Current genomic data suggest that any Western European *Canis lupus* specimen older than 30,000 years (e.g., Chauvet Cave) should be more related to wolves than to dogs. To test this hypothesis, we performed radiocarbon dating and high‐throughput DNA sequencing analysis of a Chauvet Cave coprolite morphologically identified by Garcia (2005) as canid, presumably *Canis lupus* (sample CCH3). Using global (i.e., shotgun) DNA sequencing, we obtained information on the mitochondrial and nuclear genome, and the diet of the animal.

## 2 MATERIALS AND METHODS

### 2.1 Description of Chauvet Cave coprolite CCH3

**FIGURE 2:**
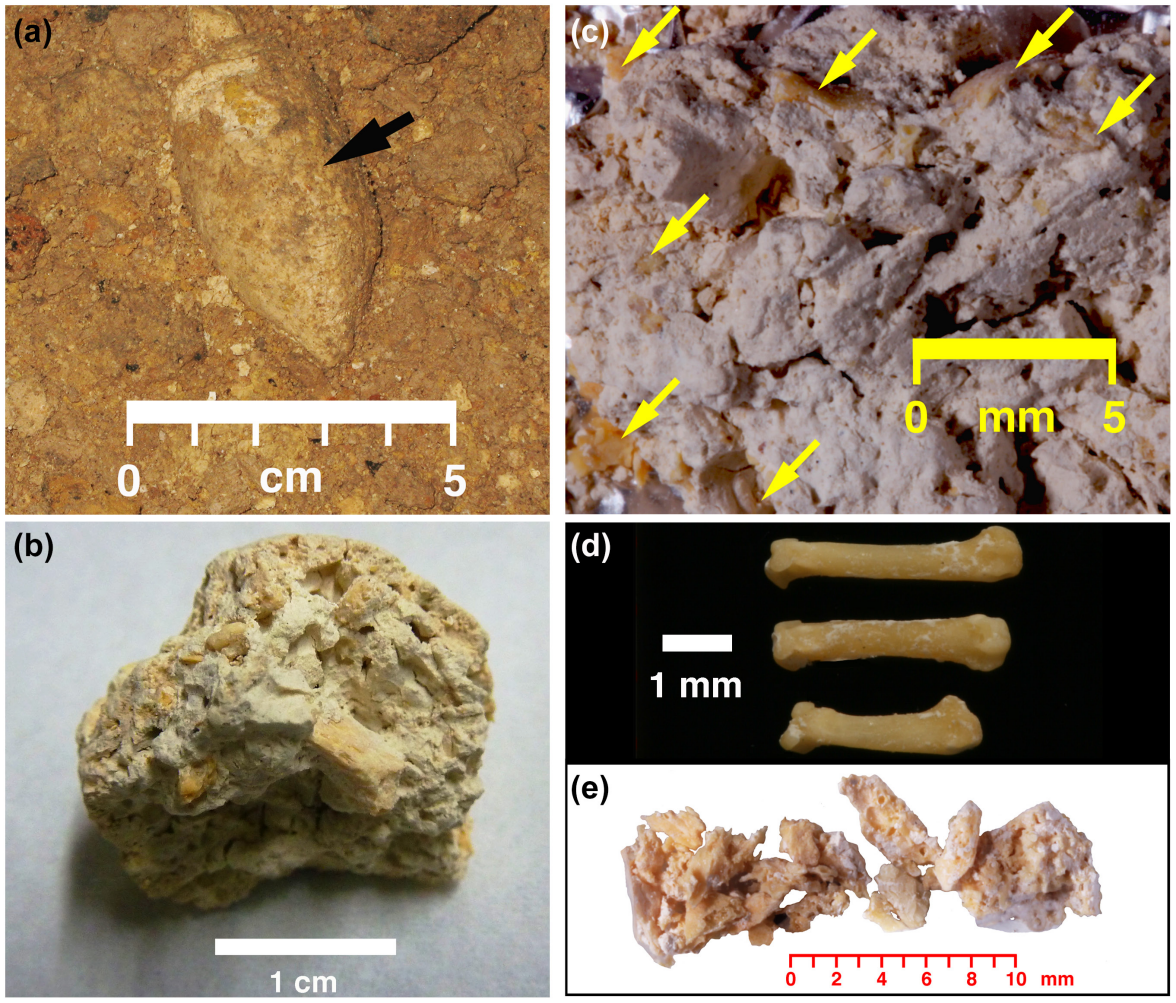
Images of Chauvet coprolite CCH3. (a) In situ coprolite CCH3 on poorly sorted, soft, friable light brown silty clay. (b) Overall photograph of coprolite's interior, white ground mass, and bone fragments ranging from 2 to 3 mm fragments to 5‐mm‐thick cortical bone fragments. (c) Close‐up of coprolite's white, amorphous ground mass and micromammal bones (yellow arrows). (d) Complete metacarpals from micromammals such as cricetids, murids, or soricids from coprolite interior. (e) 118.5 mg of cleaned bone fragments dissected from coprolite and used for AMS ^14^C dating.

Chauvet Cave coprolite CCH3 was discovered in situ during the 1997–2000 archeological investigation in Chauvet Cave (Figure 1a) Salle Hillaire, approximately 120 m from the cave's modern entrance (Figure 1b). The coprolite specimen lay on the light brown silty clay cave floor (Figure 2a). The coprolite was 5.7 cm long, 2.7 cm in diameter, weighed 22.9 g, and consisted of 95–97% white, soft, <50 μm amorphous groundmass containing 3–5% whole and fragmental bone (Figure 2b,c). Interior bone ranged from 5‐mm diameter, equant cortical fragments (Figure 2b) to complete small‐mammal metacarpals (Figure 2d) resembling cricetids, murids, or soricids, to 2‐ to 6‐mm‐long crushed small‐mammal crania (Figure 2e). Bone within the coprolite was pale yellow with a waxy luster and exhibited no obvious gastric dissolution pitting.

### 2.2 Radiocarbon dating

After manually breaking the coprolite into fragments, a 1.6 g subsample was used for AMS radiocarbon dating. 118.5 mg of bone fragments were physically dissected from the matrix (Figure 2e), washed with deionized (DI) water, decalcified with repeated changes of 0.2 N HCl for 2 days at 4°C, washed to neutrality with DI water, and treated with 0.1 M KOH overnight at 4°C to remove humates. Gelatin was extracted by heating the alkali‐extracted, demineralized collagen at 90°C in 0.05 N HCl. Complete gelatinization in 0.05 N HCl occurred after 20 min rather than the several hours commonly required for fossil collagens. After filtering the gelatin solution through a 0.45 μm Durapore filter membrane, the filtrate was passed through a 30,000 Dalton (30 kDa) membrane, and the >30 kDa was used for ^14^C analysis. This >30 kDa fraction was freeze‐dried, weighed into a 4 mm ID quartz tube, evacuated to <10 mTorr under LN pumping, sealed with an H_2_/O_2_ torch, and the sealed, evacuated tube combusted at 850°C for 1 h. CO_2_ was collected cryogenically, converted into graphite using the H_2_–Fe method (Vogel et al., 1984), and dated by AMS ^14^C at the Aarhus AMS Centre (AARAMS) at Aarhus University, Denmark. The calibrated age of the sample, expressed in calendar years before present (cal BP), was calculated using OxCal 4.4 software (Ramsey, 2009) and the IntCal 20 calibration curve (Reimer et al., 2020).

### 2.3 DNA extraction

Pre‐PCR steps, i.e., DNA extraction and construction of DNA libraries, were performed in laboratories dedicated to ancient DNA studies in CEA Saclay (Bon et al., 2008) and Musée de l'Homme (Utge et al., 2020). Before DNA extraction, the exterior ~0.5 mm of Chauvet coprolite CCH3 was removed with a single‐use surgical blade. We performed two DNA extractions from the CCH3 coprolite. One extraction used material from both the cleaned exterior surface and the core (center) of the coprolite and is referred to as sample CCH3T. The second extraction used material only from the core of the coprolite; its corresponding extract is labeled CCH3C. DNA extraction was performed as in Palacio et al. (2017), with 1 g of coprolite being pulverized with a Mikro‐Dismembrator S (Sartorius; Goettingen, Germany) set to a shaking frequency of 2000/min for 30 s, transferred into 10 ml of DNA extraction buffer (0.45 M EDTA, 10 mM Tris–HCl [pH 8.0], 0.1% SDS, 65 mM DTT, 0.5 mg/ml proteinase K), and incubated 16 h at 42°C under constant agitation. After centrifugation, the supernatant was recovered, extracted once with one volume of phenol, once with a phenol‐chloroform (50:50) mixture, and once with chloroform. The aqueous phase was dialyzed and concentrated using a 2 ml centrifugal ultrafilter with a 30 kDa cutoff (Millipore, Billerica, MA). The column was washed four times with distilled water, and the DNA extract was recovered in a volume of 100–120 μl. The DNA extract was purified further using a silica‐membrane‐based method (Qiagen purification kit #28004; Venlo, Netherlands). The final extract was eluted from the column with 100 μl of TE buffer.

### 2.4 Real‐time PCR studies

Real‐time PCR analysis used TaqMan MGB probes from Thermo Fisher Scientific. Primers and probes for the TaqMan assays (Table A1 in Appendix) were designed with the help of Primer Express software 3.0.1 (Thermo Fisher Scientific) using complete mitochondrial genome sequences of *Canis lupus* (DQ480505), *Ursus spelaeus* (EU327344), *Ursus arctos* (EU497665), *Martes foina* (HM106325), *Crocuta crocuta* (JF894378), *Coelodonta antiquatis* (FJ905813), and *Cervus elaphus* (AB245427). Real‐time PCR was carried out in a 20‐μl reaction volume containing 10 μl of 2X TaqMan fast advanced master mix (Thermo Fisher Scientific), 900 nM of forward and reverse primers, 250 nM of TaqMan probe, and DI water (PCR blanks), mock or DNA extracts. For the Chauvet Cave coprolite, two 0.5‐μl replicate samples of the CCH3 core DNA extract were analyzed using all seven TaqMan assays. For control samples, four of which have been published previously (Bon et al., 2008; Bon et al., 2012; Utge et al., 2020), two 0.05‐μl or 0.5‐μl replicate samples of the DNA extracts were analyzed. Amplification was performed in a CFX96 Touch thermocycler (Bio‐Rad, Hercules, CA, USA) and included the following steps: 50°C, 2 min; 95°C, 20 s; then 40 PCR cycles (95°C, 3 s; 60°C, 30 s). Data were analyzed using the Bio‐Rad CFX Maestro software set with default parameters to determine the cycle threshold (*C*_T_), i.e., the number of PCR cycles required for the fluorescent signal to exceed the background.

### 2.5 Comparative PCR analysis of cave bear DNA in sediments and in the CCH3 coprolite

DNA extraction was carried out from 1 g of sediments using the method described above for the coprolite, and the DNA extract was eluted from the Qiagen column with 100 μl of TE buffer. The presence of cave bear DNA in sediments and in the coprolite CCH3T extract was analyzed using primers designed to amplify efficiently a 112‐bp fragment of the 16S mitochondrial ribosomal gene (Bon et al., 2008). The primers (forward primer: 5′‐AACAACATATTCCTTCCATGAGC‐3′; reverse primer: 5′‐AAGTAAGTGAGCATTTTGACTGGTAC‐3′) encompass a fragment of the mitochondrial genome where the cave bear and brown bear sequences display 5 mismatches. PCR was carried out in a 50‐μl reaction volume containing 300 nM of forward and reverse primers, 200 μM dNTP, 2.5 mM MgCl_2_, 5 μl of GeneAmp AmpliTaq Gold DNA polymerase buffer II, 2.5 U of AmpliTaq Gold DNA polymerase (Thermo Fisher Scientific), and variable amounts (1–10 μl) of the sediments DNA extract, 5 μl of mock extract, or 0.5 μl of the CCH3T DNA extract. PCR procedures consisted of an enzyme activation step (95°C, 10 min), followed by a single round of 45 PCR cycles (95°C, 15 s; 53°C, 20 s; 70°C, 1 min) performed in a Veriti thermal cycler (Thermo Fisher Scientific). To detect PCR amplicons, the full reaction volume was loaded onto an 8% polyacrylamide gel stained with Sybr Green I (Thermo Fisher Scientific). To confirm the amplification of cave bear DNA, we performed Sanger DNA sequencing of cloned amplicons as described (Bon et al., 2008).

### 2.6 Generation and sequencing of DNA libraries

Libraries of DNA fragments suitable for high‐throughput sequencing on the Illumina platform (Bentley et al., 2008) were generated using 5–10 μl of CCH3T or CCH3C DNA extracts and Illumina reagents (Table A2 in Appendix). The coprolite or mock DNA extract was 5′end‐phosphorylated and blunt‐ended using a mixture of T4 polynucleotide kinase, T4 DNA polymerase, and Klenow DNA polymerase; the reaction product was purified on a Qiagen 28004 column. Second, a 3′ adenine residue was added to the blunt‐ended DNA fragments using Klenow 3′ to 5′ exo‐ polymerase, and the product was purified on a Qiagen 28004 column (libraries 1–4), or the enzyme was heat‐inactivated at 70°C (library 5). Third, Illumina adapters with overhanging thymine were ligated to the DNA fragments; the reaction product was purified either on an 8% polyacrylamide gel (libraries 1–2) or a Qiagen 28104 column (libraries 3–5) and recovered in a volume of 30 μl. Fourth, 30%–100% of each library was PCR‐amplified (12 PCR cycles) from 1 to 6 replicate samples using either Phusion or Phusion U DNA polymerase (Table A2 in Appendix). Fifth, the full PCR reaction volume was loaded onto an 8% polyacrylamide gel stained with SYBR Green I; fragments containing a 30–100‐bp insert (in addition to the DNA derived from the adapters) were cut from the gel and purified. No signal other than the amplification of the adapters was obtained for libraries produced from mock extracts.

DNA sequencing was performed at Genoscope (CEA Evry, France) using Illumina GaIIx or HiSeq platforms with a read length set to 76 to 101 nucleotides and analysis on the single read mode.

### 2.7 Processing of DNA reads

Reads were trimmed for adapter sequences, N's, and low‐quality stretches on the 3′ end, using a software based on the FASTX‐Toolkit package (http://hannonlab.cshl.edu/fastx_toolkit) and designed by Genoscope. Only DNA reads ≥30 nucleotides were considered for the analysis.

### 2.8 Analysis of mitochondrial DNA reads

To identify the animal from which the coprolite originated and gain insight into that individual's diet, DNA reads were aligned simultaneously by competitive mapping (Palacio et al., 2017) to a set of 35 mitochondrial genomes, including the dog (CanFam3.1) reference mitochondrial genome sequence. This set of genomes was successfully used in previous studies to gain insight into a carnivore diet from Pleistocene coprolites (Elalouf et al., 2021). The alignment was performed using BWA version 0.7 (Li & Durbin, 2009) with the *aln* option recommended for ancient DNA, including disabled seed (−l 1024) and permissive parameters (−n 0.01 −o 2; Schubert et al., 2012, Martiniano et al., 2020). DNA reads with a mapping quality lower than 25 were removed using Samtools (Li et al., 2009) and duplicate reads were discarded using Picard MarkDuplicates (http://broadinstitute.github.io/picard/). Mapped reads were re‐aligned through a BLAT‐based alignment onto the reference panel derived from the 35 species using stringent criteria (‐minScore = 30 ‐minIdentity = 90) and only reads with a scoring parameter ≥95% were retained.

### 2.9 Reconstruction and phylogenetic analysis of the canine mitochondrial genome sequence

**FIGURE A1:**
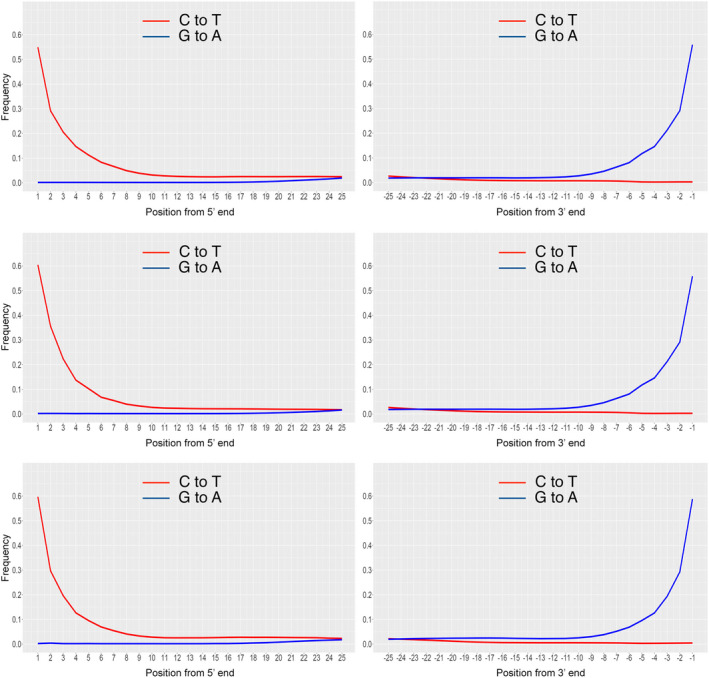
Patterns of DNA damage in libraries amplified using Phusion U DNA polymerase. The figure shows positions of the mismatches in DNA reads used to reconstruct the *Canis lupus* mitochondrial genome sequence for library BXW_COSU (upper panel), BXW_BOSU (middle panel), and BXW_AOSW (lower panel). Substitution frequencies, calculated by comparing individual reads to the dog reference mitochondrial genome sequence (CanFam3.1), are plotted as a function of the distance from the 5′ end or the 3′ end of the reads.

**FIGURE A2:**
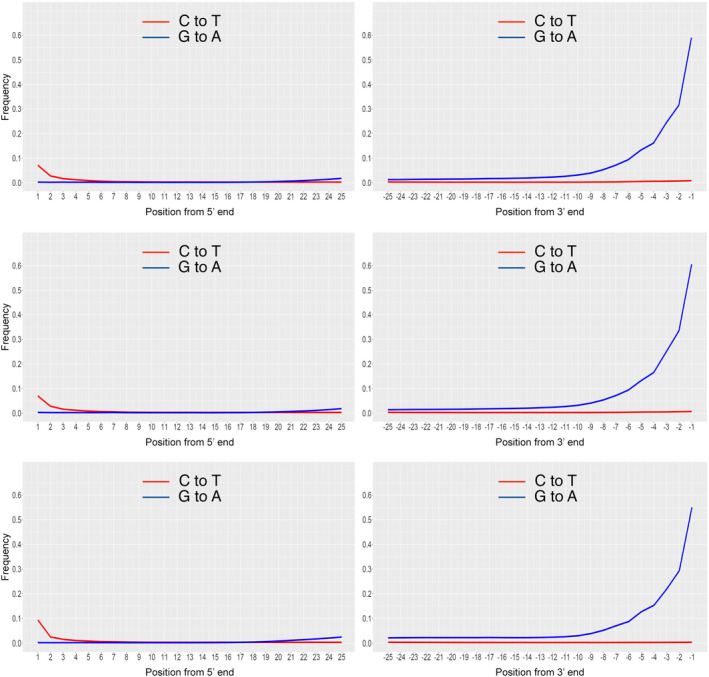
Patterns of DNA damage in libraries amplified using Phusion DNA polymerase. The figure shows positions of the mismatches in DNA reads used to reconstruct the *Canis lupus* mitochondrial genome sequence for library AQG_BOSS (upper panel), AQG_COSS (middle panel), and AQG_GOSU (lower panel). Substitution frequencies, calculated by comparing individual reads to the dog reference mitochondrial genome sequence (CanFam3.1), are plotted as a function of the distance from the 5′ end or the 3′ end of the reads.

Because the competitive mapping studies demonstrated that more than 99% of mitochondrial DNA reads corresponded to canid DNA, we undertook to reconstruct a complete canid mitochondrial genome sequence by aligning the reads to the CanFam3.1 reference genome. DNA reads were aligned to this genome using BWA with the *aln* algorithm as described above. Only reads with a mapping quality ≥25 were considered. Mapped reads analyzed using mapDamage 2.0 (Jónsson et al., 2013) displayed the expected pattern of nucleotide misincorporation for ancient DNA sequences, with inflated CG to TA substitution rates toward reads ends in libraries generated using Phusion U DNA polymerase (Figure A1 in Appendix); conversely, in libraries generated using Phusion DNA polymerase (which stalls polymerization upstream of deaminated cytosine) only inflated G to A substitution rates were observed toward 3′ ends (Figure A2 in Appendix).

For phylogenetic analysis, the consensus mitochondrial sequence derived from the DNA reads was aligned to a set of mitochondrial genomes using Clustal‐Omega 1.2.4 (Sievers & Higgins, 2021). Initial analyses were carried out using the Thalmann et al. (2013) dataset of ancient and modern wolves and dogs. They demonstrated that the Chauvet mitochondrial genome sequence positions close to the genomes of ancient wolves, well apart from the phylogenetic position of ancient and modern dog genomes (Palacio, 2015). Subsequent analyses were therefore conducted using a large dataset of complete wolf mitochondrial genome sequences, of which 45 are from ancient and 90 are from modern specimens (Loog et al., 2020). We used BEAST v.1.8.0 software (Drummond et al., 2012) to build a tip‐calibrated wolf mitochondrial tree. The ages (in calendar years before present) of the ancient samples in BEAST analysis were set as published in Loog et al. (2020). The geologic age of the Chauvet coprolite was set as 35,000 cal BP, based on the sample's calibrated radiocarbon date. All input files for BEAST analysis were created using BEAUti v.1.8.0 (Drummond et al., 2012) with default parameters. Priors were used as specified in the Loog et al. (2020) supplementary data. The posterior distribution and divergence times of nodes were estimated by Markov Chain Monte Carlo (MCMC), where model parameters and trees were sampled every 1000 iterations over 10,000,000 iterations, following a discarded burn‐in of 1 million iterations. The MCMC chain convergence for all parameters was assessed using Tracer v1.7 (Rambaut et al., 2018); the sampled trees were summarized, and the maximum clade credibility tree was calculated using TreeAnnotator v1.8.0 (Drummond et al., 2012). The tip‐calibrated BEAST tree of all samples was plotted using R packages ggplot2 ggtree.

### 2.10 Nuclear genome analysis

#### 2.10.1 Genome coverage and sex determination

Nuclear genome coverage was evaluated using reads of 30 nucleotides or more that aligned to the CanFam 3.1 dog reference genome with a mapping quality and base quality of at least 25. To determine the animal's sex for the Chauvet specimen, we compared the coverage of the X and autosomes and calculated the ratio of the X and autosomes coverages. Calculation of the heterozygosity was performed using runSexDetramination.sh tool (https://github.com/stschiff/GAworkshop/blob/master/contents/03_sexdet/sexdet.rst).

#### 2.10.2 Genotypes and SNP calling

Genotypes were called using reads of 30 nucleotides or more that aligned to the CanFam 3.1 dog reference genome with a mapping quality and base quality of at least 25 at each covered position in the genome. SNPs were called using bcftools and filtered using vcftools (Danecek et al., 2011). We used bcftools ‘mpileup’ v0.1.19 (Li, 2011) to call genotypes with default settings. Data were filtered using highly stringent criteria, including the exclusion of the first and last 5 nucleotides of each read, the selection of positions covered by at least 10 reads, and the removal of SNPs consisting of nucleotide transitions.

#### 2.10.3 Dataset used for comparative studies

We used the “722 g” variant calling format (VCF) file containing genotypes called from whole‐genome sequencing data for 722 specimens. This file includes genome data for modern dogs, wolves, and other related canid species compiled by the National Human Genome Research Institute dog genome project (Plassais et al., 2019; BioProject: PRJNA448733). The 722 g VCF containing more than 90 million SNPs and indels was filtered as indel alleles were removed by setting the genotype of any individual carrying such an allele to missing (thereby retaining any overlapped SNP alleles). The genotypes carrying any allele other than the two most common alleles were set to missing (thereby retaining only the two most common alleles at multi‐allelic sites).

We mostly used the population labels and descriptions of modern genomes from the 722 g VCF metadata; however, we have modified some labels for consistency. For example, “Wolves” were grouped as New World and Old World; other canids, consisting of coyote (*Canis latrans*), golden‐jackal (*Canis aureus*), red wolf (*Canis rufus*), dhole (*Cuon alpinus*) and Andean fox (*Lycalopex culpaeus*) were grouped together. We selected a subset of 104 specimens (Table A3 in Appendix) of the 722 g dataset. All wolves (*N* = 46), all other canids (*N* = 8), and the Dingo (*Canis lupus dingo*, *N* = 1) were considered; for dogs, because the unbalanced representation of ancestries can distort the results of analyses such as PCA, we considered subpopulations based on their geographical origin from which we randomly selected a subset of 49 *Canis lupus familiaris* specimens from around the world. The genotypes of the ancient Chauvet coprolite were merged into this VCF using bcftools isec, yielding a dataset of 241,307 SNPs.

#### 2.10.4 Phylogenetic analysis

We used PLINK (Purcell et al., 2007) to compute an identity by state (IBS) matrix. Then, phylogenetic analysis was performed using the Neighbor‐Joining method with the help of the R package “ape” (Paradis & Schliep, 2019), and the robustness of the phylogenetic inferences was estimated using the bootstrap method (1000 replicates).

#### 2.10.5 Population structure: PCA and admixture analysis

Identifying variant sites and calling genotypes have been shown to introduce biases when dealing with a low‐coverage draft genome (Korneliussen et al., 2014). To avoid these issues, we performed the analyses using genotype likelihoods (Hui et al., 2020) that were computed in ANGSD v0.921 (Korneliussen et al., 2014) using bam files from the Chauvet ancient genome and the genotype posterior probabilities (Browning & Yu, 2009) in the Beagle format from modern genomes.

We used PCAngsd (Meisner & Albrechtsen, 2018) and NGSadmix (Skotte et al., 2013) on the genotype likelihoods computed in ANGSD for PCA and to evaluate admixture proportions in our dataset. For Principal Component Analysis, the Chauvet sample and a set of 104 samples including 49 modern dogs, 46 modern wolves, the Dingo, and 8 other canids were considered (see above). We explored the population structure by running the admixture analyses on the Chauvet sample and 34 wolves representative for Old World (excluding wolves with undefined country of origin) and New World specimens, with a different number of estimated ancestry clusters, ranging from *K* = 2–5.

## 3 RESULTS AND DISCUSSION

### 3.1 AMS radiocarbon dating of Chauvet Cave coprolite CCH3

**FIGURE 3:**
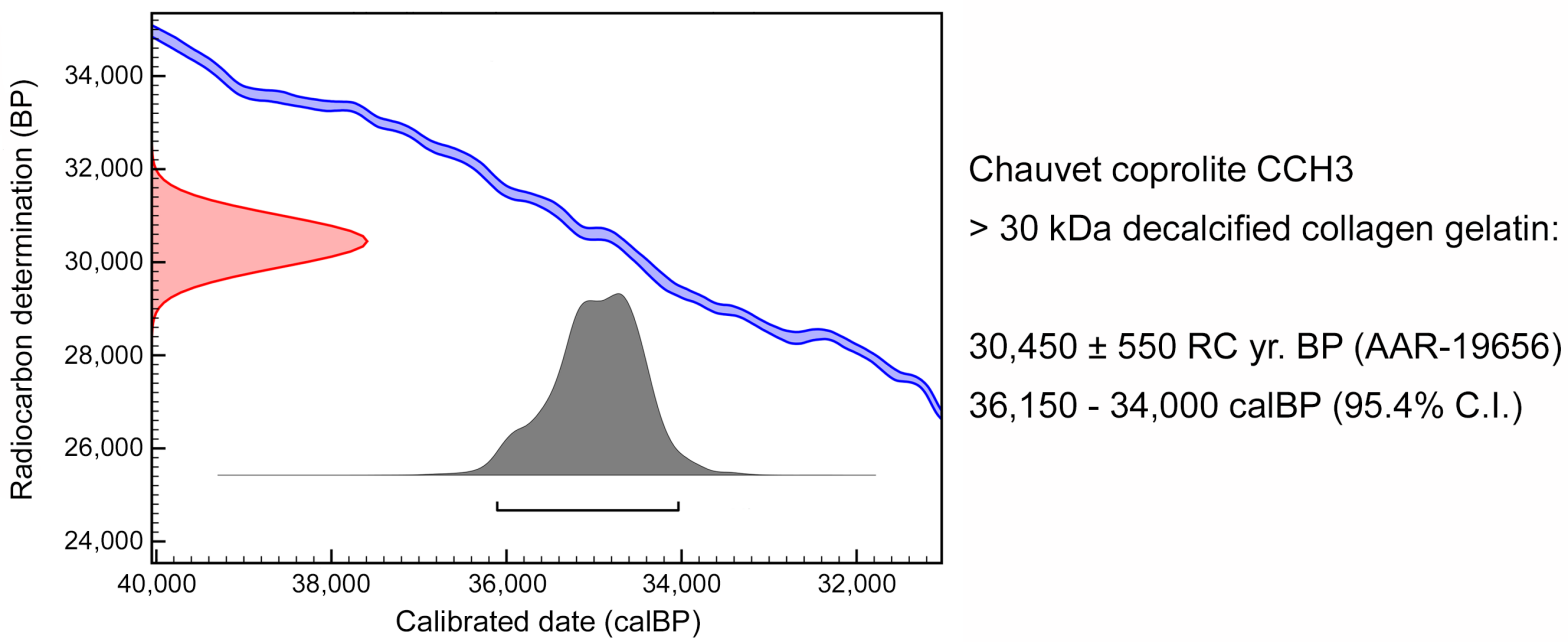
Uncalibrated (BP) and calibrated (cal BP) age of the ultrafiltration‐purified (>30 kDa) gelatin from bone fragments shown in Figure 2e. The cal BP data correspond to 95.4% (2σ) confidence interval of the sample age determined using OxCal v4.4 software and IntCal20 calibration curve.

Radiocarbon dating was performed using bone fragments isolated from the coprolite. The measured age was 30,450 ± 550 RC yr. BP (AAR‐19656; 36,150–34,000 cal BP; 95.4% probability; Figure 3). This age is similar to the ones obtained for several cave bear remains from the Chauvet Cave (Quiles et al., 2016).

### 3.2 Mitochondrial DNA reads allow for the identification of the defecator and of the animal's diet

**FIGURE 4:**
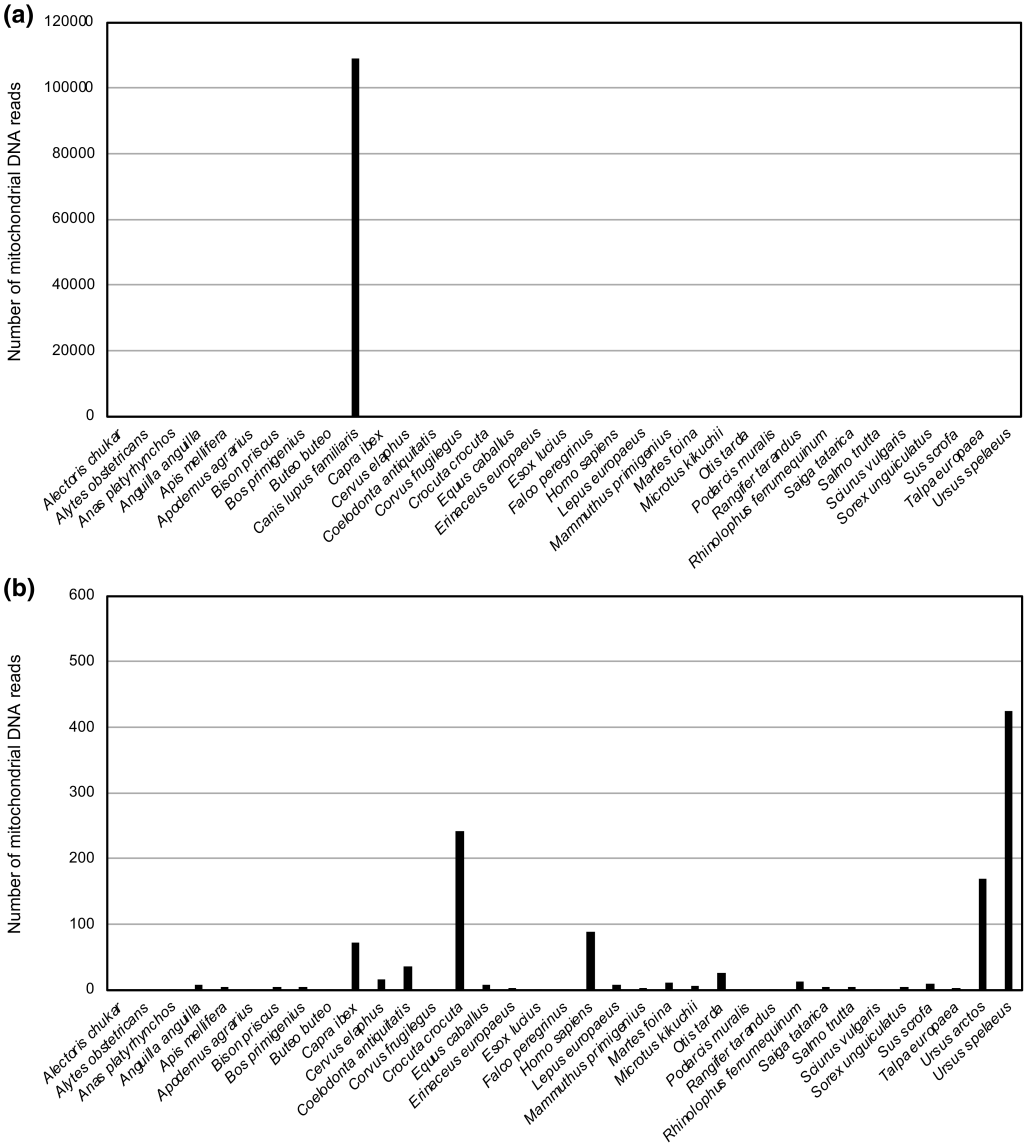
Number of reads of the Chauvet coprolite DNA libraries aligned to the reference mitochondrial genome sequence of the indicated animal species. (a) Alignment to the mitochondrial genome of *Canis lupus familiaris* and 34 other animal species demonstrates the predominance of *Canis lupus familiaris* DNA reads. (b) Enlarged *Y*‐axis scale (without *Canis lupus familiaris* data). Results of the alignment to the *Ursus arctos* genome are shown and indicate that a much higher number of reads align to *Ursus spelaeus* than to *Ursus arctos*.

Results of alignments of the DNA reads to the complete mitochondrial genome sequence of 35 species show that more than 99% (>100,000) of the mapped reads aligned to the dog reference CanFam3.1 mitochondrial genome, whereas <1000 reads aligned to each of the other genomes (Figure 4a). These results are an indication that a canid is the most probable source of the feces. Further examination of the data (Figure 4b) revealed that several hundred reads aligned to the genomes of two species from the Mammalian order Carnivora – the extinct cave bear (*Ursus spelaeus*) and the extinct cave hyena (*Crocuta crocuta*). Less than 100 reads aligned to genomes of all other species, including cricetid (*Microtus kikuchii*), murid (*Apodemus agrarius*), and soricid (*Sorex unguiculatus*) species considered because of the bone coprolite content (see Methods).

**FIGURE 5:**
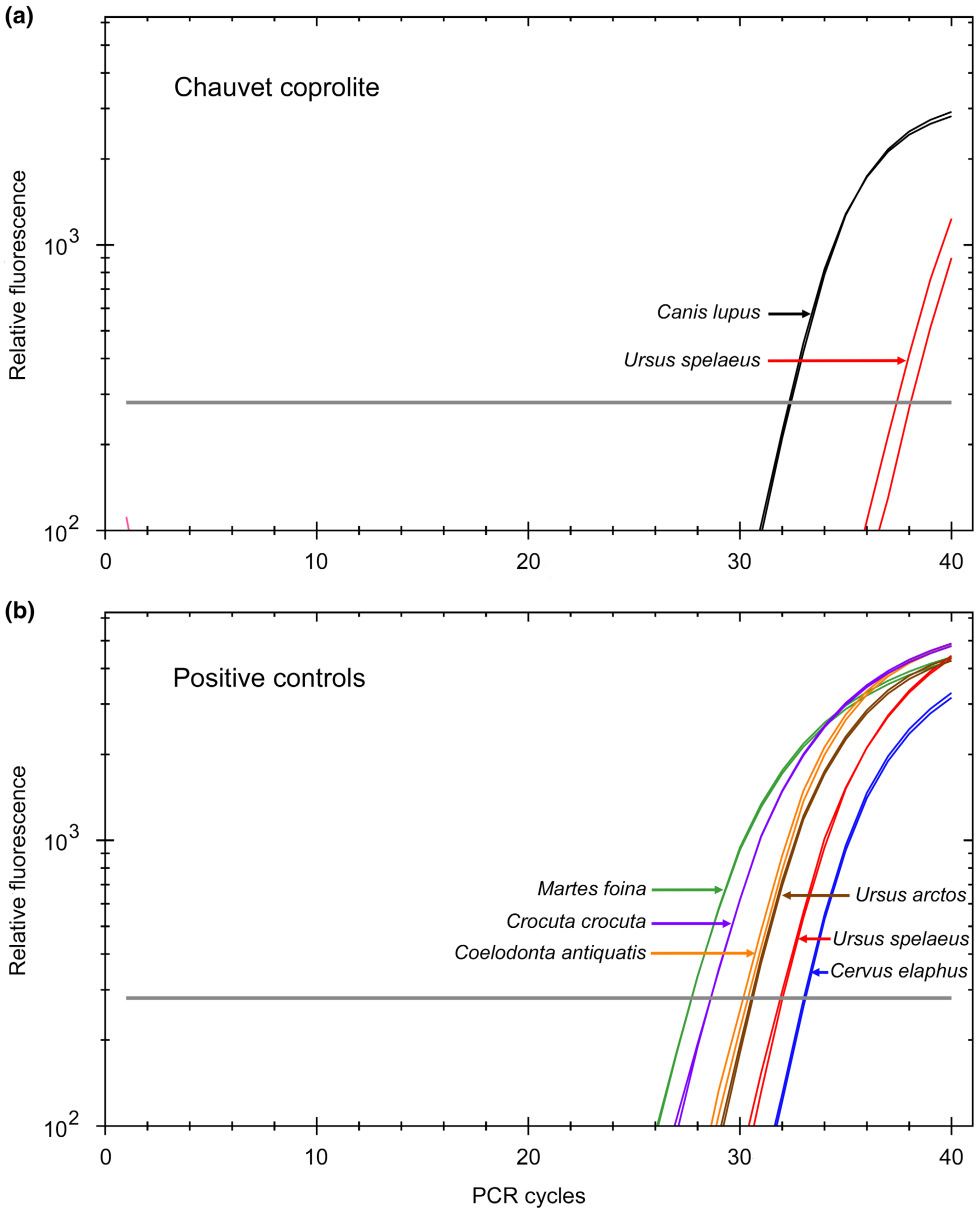
Real‐time PCR studies. (a) Analysis of the Chauvet Cave coprolite. The figure shows that the *Canis lupus* and *Ursus spelaeus* PCR TaqMan assays allowed to demonstrate the presence of DNA for each of these two species in the coprolite. The gray horizontal line indicates the fluorescence value considered for the determination of cycle threshold (*C*_T_) amplification values. Amplification signals for all other tested species (*Martes foina*, *Crocuta crocuta*, *Coelodonta antiquatis*, *Ursus arctos*, *Cervus elaphus*), blank and mock extracts were not significant. (b) Validation of the PCR TaqMan assays for the indicated species using positive control samples.

**FIGURE A3:**
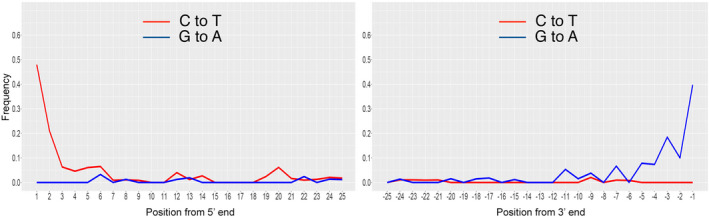
Patterns of damage in cave bear DNA reads from CCH3 coprolite libraries. Substitution frequencies, calculated by comparing individual reads to the *Ursus spelaeus* complete mitochondrial genome sequence (Bon et al., 2008), are plotted as a function of the distance from the 5′ end or the 3′ end of the reads. Because of the low number of unique cave bear DNA reads (*N* = 424), data from all libraries were pooled. Hence the patterns of damage, intermediate to those depicted in Figures A1 and A2 for C to T substitutions.

The number of reads aligned to *Ursus spelaeus* was found significant (Grubbs test, *p*‐value = 5.48e−09) when compared with reads aligned to other genomes, and the pattern of damage of these reads is consistent with that expected for ancient DNA (Figure A3 in Appendix). However, since both the extinct cave bear *Ursus spelaeus* and the brown bear (*Ursus arctos*) were present in France 35,000 years ago, we performed competitive mapping using the brown bear mitochondrial genome. With a higher number of reads aligned to the *Ursus spelaeus* than to the *Ursus arctos* genome (Figure 4b), the data are an indication that the Chauvet canid had eaten cave bear rather than brown bear. This conclusion is strengthened by real‐time PCR studies carried out using specific TaqMan assays. As anticipated from the shotgun DNA sequencing data, we obtained robust amplification of *Canis lupus* DNA and also detected *Ursus spealeus* DNA in the coprolite extract (Figure 5a). On the other hand, no amplification signal was obtained for the other species investigated, including *Ursus arctos*, *Crocuta crocuta*, *Martes foina*, *Coelodonta antiquatis*, and *Cervus elaphus*, even though the corresponding TaqMan assays were validated using positive controls (Figure 5b).

**FIGURE 6:**
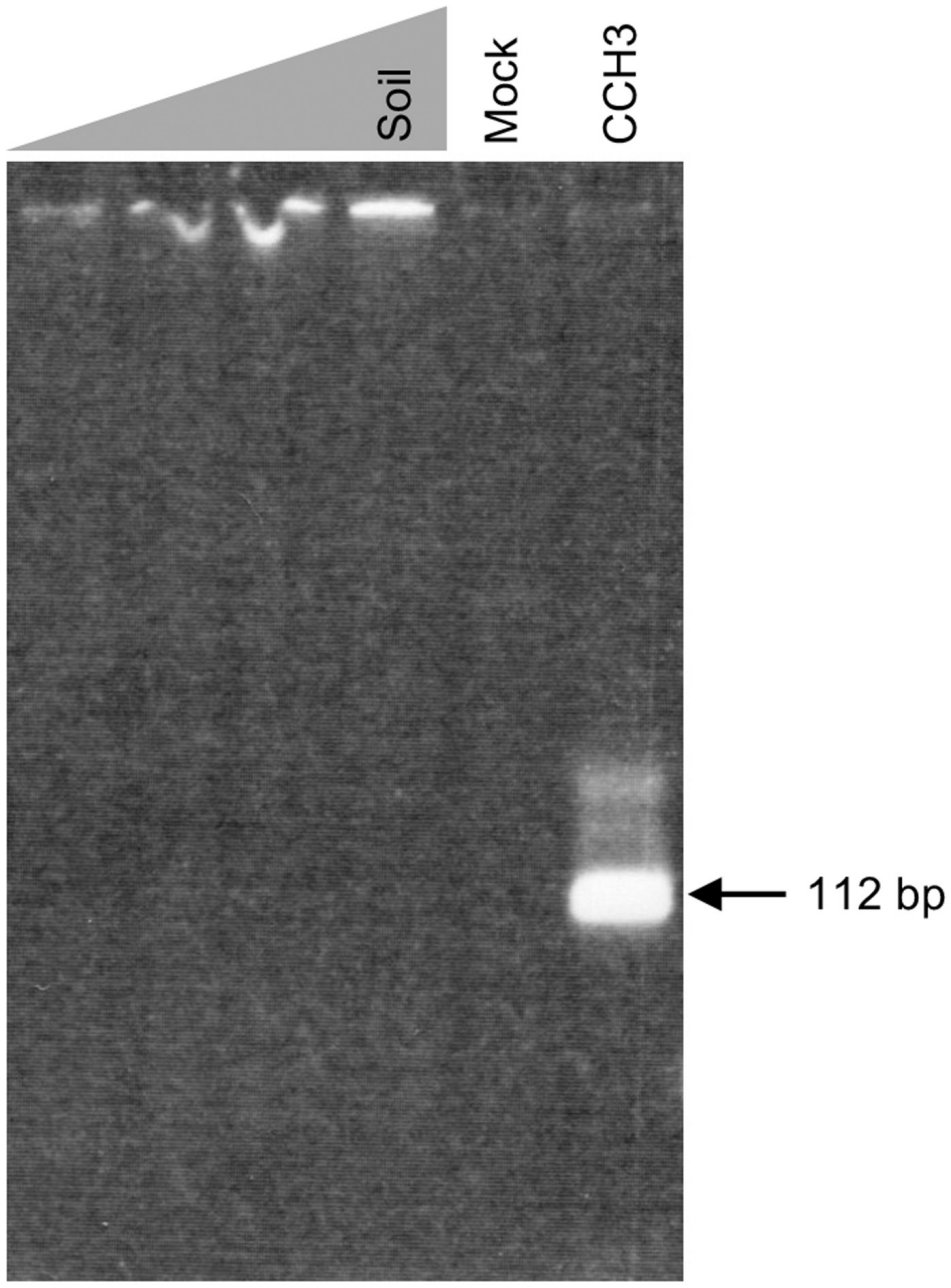
Comparative PCR analysis of the CCH3 Chauvet Cave coprolite and of a soil sample from the same cave sector. The figure shows gel electrophoresis analysis of PCR products obtained from the soil DNA extract, a mock extract, and the coprolite DNA extract using primers designed to amplify a 112‐bp fragment of the cave bear mitochondrial genome. The experiment was performed using variable amounts (1–10 μl) of the soil DNA extract and 0.5 μl of the coprolite DNA extract. Note that successful amplification was only obtained from the CCH3 coprolite DNA extract. The identity of the PCR product was confirmed by DNA sequencing.

Considering the large number of cave bear bones in the Chauvet Cave, we also explored the possibility that the presence of cave bear DNA in the coprolite might originate from a taphonomic process. For this, we performed comparative PCR studies of the coprolite and of a soil sample from the same sector. These studies provided evidence for cave bear DNA in the coprolite but not in the soil sample (Figure 6), indicating that such DNA highlights the diet rather than some leakage process from the environment.

Overall, the shotgun sequencing and PCR data both demonstrate the presence of large amounts of mitochondrial canid DNA in the 35,000‐year‐old Chauvet coprolite, supporting the conclusion that its origin is an ancient canid. Small amounts of *Ursus spelaeus* mitochondrial DNA are also detected by shotgun DNA sequencing and PCR analysis, indicating that the extinct cave bear was part of the diet of the Chauvet canid. The lack of evidence for the presence of cricetid, murid, or soricid DNA is likely due to the small amount of tissue ingested from such species as compared to the large mass of cave bear tissue eaten. At any rate, this indicates that, in addition to ancient DNA studies, all sources of information are of interest to characterize a coprolite sample.

### 3.3 Reconstruction and phylogenetic analysis of a complete *Canis lupus* mitochondrial genome sequence

**FIGURE 7:**
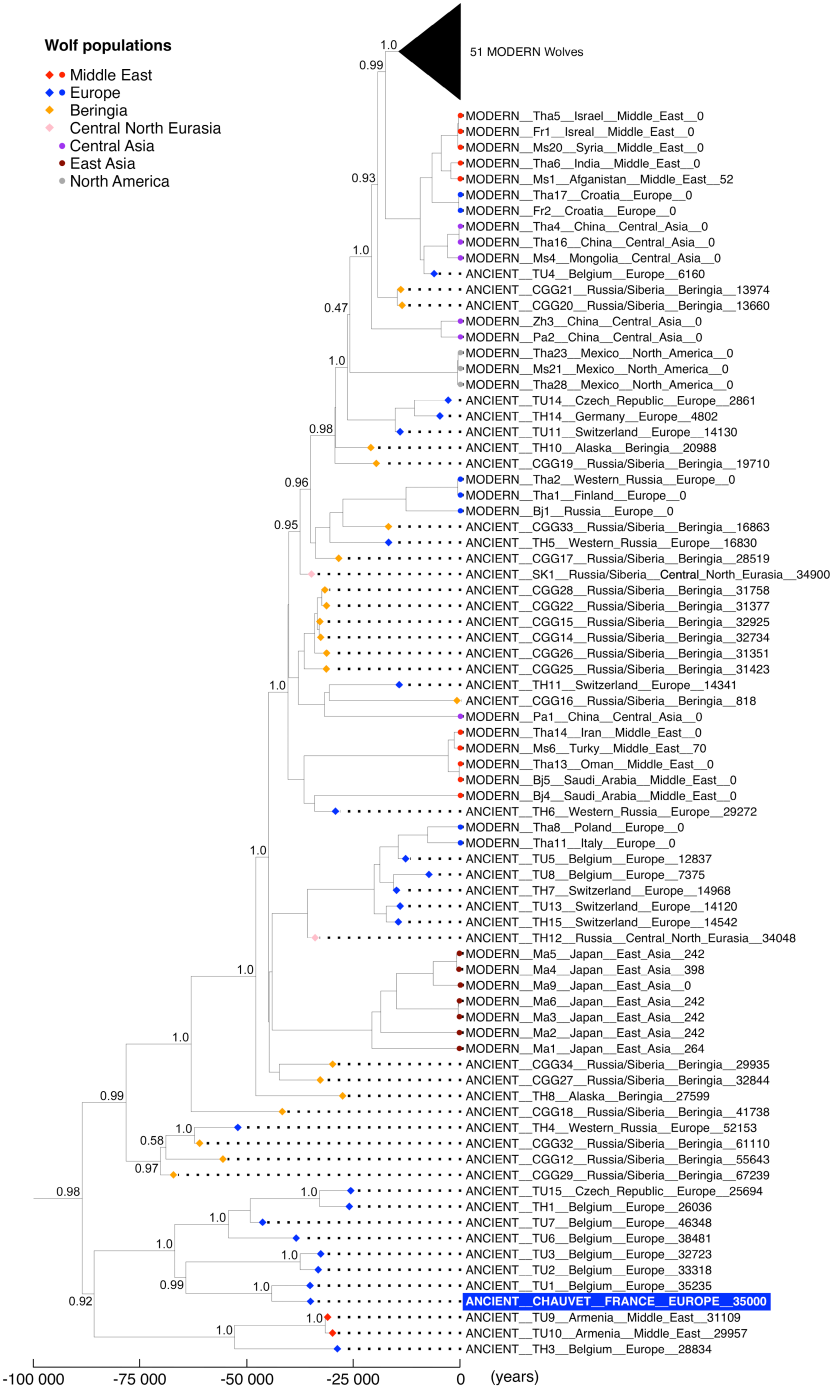
Phylogenetic position of the Chauvet mitochondrial canid genome sequence. The figure shows the tip calibrated BEAST tree obtained using the canid sequence reconstructed from Chauvet coprolite DNA and a dataset of 135 (90 modern, 45 ancient) complete wolf mitochondrial genomes from across the Northern hemisphere (Loog et al., 2020). Values reported adjacent to the name and geographic origin of each specimen correspond to ages used for tip calibration. Diamonds represent ancient (<500 years) samples, and circle represents modern samples. The tree was rooted using modern Tibetan wolves, not shown in the figure. Posterior probabilities are given for major nodes. The cluster of 51 modern wolves includes wolves from North America (*N* = 13), Arctic North America (*N* = 10), Beringia (*N* = 11), Europe (*N* = 11), Middle East (*N* = 2), East Asia (*N* = 2), Central Asia (*N* = 1), and Central North Eurasia (*N* = 1).

The large number of mitochondrial canid DNA reads obtained from the coprolite enabled us to reconstruct a nearly complete mitochondrial genome sequence (83× average unique read depth; total number of gaps: 48 nucleotides). We performed a phylogenetic analysis of the Chauvet mitochondrial genome by comparing its sequence to those of 135 previously published complete mitochondrial wolf genome sequences (Loog et al., 2020) obtained from 45 ancient and 90 modern specimens. As pointed out by Loog et al. (2020), a group of ancient samples from Europe and the Middle East delineates a divergent clade (Figure 7). The most recent common ancestor (MRCA) of specimens from this clade and all other ancient or modern wolves dates to ~90,000 years ago. The Chauvet coprolite's mitochondrial genome sequence belongs to this clade. Interestingly, the Chauvet sequence displays the closest phylogenetic relationship with the TU1‐TU3 specimens, all of which date to the same time period (32,700–35,000 cal BP) and all originate from Trou‐Magritte cave (Belgium), 700 km north of Chauvet Cave. This observation supports the hypothesis that a European population of ancient wolves has not contributed to the present‐day mitochondrial diversity of wolves (Loog et al., 2020). Most other ancient samples are scattered in a distinct clade that comprises specimens from both ancient different time periods and also modern wolves from across the Northern Hemisphere. The age of the four samples that fall at the base of this clade (CGG12, −29, and −32: Eastern [Siberia] Russia; TH4: Western Russia) has been estimated by molecular dating (Loog et al., 2020).

### 3.4 Nuclear genome analysis

#### 3.4.1 Chromosome coverage and sex determination

**FIGURE 8:**
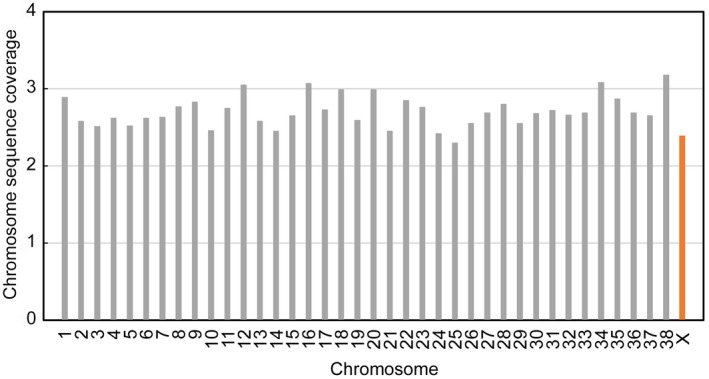
Coverage of the *Canis lupus familiaris* reference nuclear genome (CanFam3.1). The figure shows the average coverage of each chromosome by DNA sequence data obtained from the Chauvet coprolite.

We performed a genome‐wide analysis of the Chauvet canid nuclear genome by mapping the DNA reads on the dog reference genome (CanFam3.1). Over 194 million unique reads equal to or greater than 30 nucleotides were mapped, confirming the high content of canid DNA in the coprolite. The coverage of the autosomes ranged between 2.30 and 3.18 (Figure 8) and averaged 2.71 ± 0.20 (mean ± SD). The coverage of the X chromosome (2.39) was not significantly different from this value. Furthermore, the calculation of the heterozygosity showed that the heterozygosity rate of the X chromosome was comparable to the 38 autosomes (range 36–42%). We conclude from the chromosome coverage and the heterozygosity analysis that the feces were produced by a female individual.

#### 3.4.2 Comparison of Chauvet and extant canids nuclear genomes

To call variants, we used the bcftool genotype caller to generate 241,307 variant sites with a depth ≥ 10. Considering transversions variant sites, we analyzed the Chauvet coprolite within the context of a comprehensive collection of 722 modern canid whole genomes sequenced at medium to high coverage, including breed dogs, village dogs, wolves, and wild canids from which a subset of 104 samples was selected (see Methods and Table A3 in Appendix).

**FIGURE 9:**
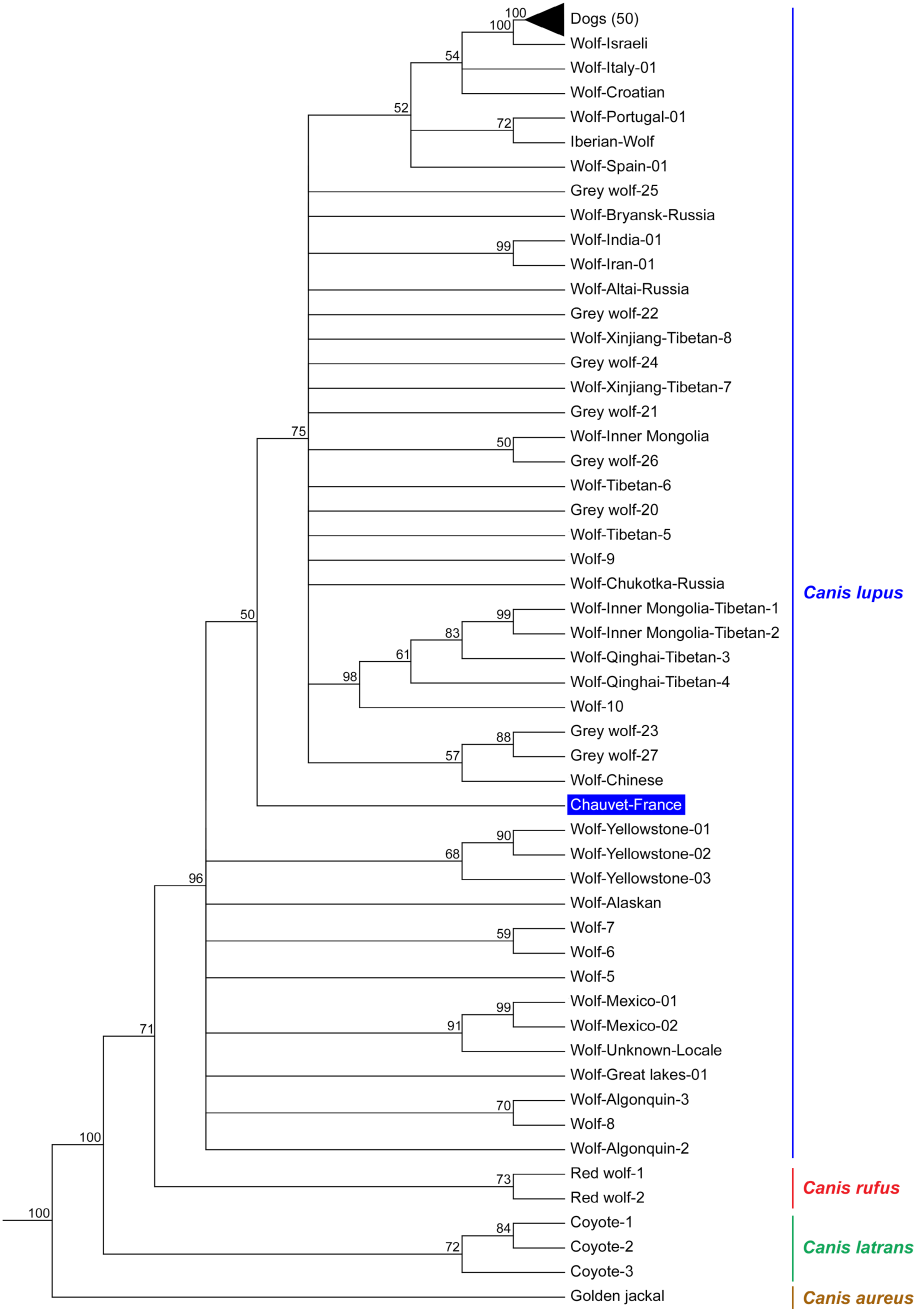
Phylogenetic position of the Chauvet canid specimen inferred from nuclear sequence data. Tree construction was performed by Neighbor‐Joining (NJ) analysis, using the Andean fox (*Lycalopex culpaeus*) and the dhole (*Cuon alpinus*) to root the tree. The robustness of the tree was evaluated using the bootstrap method (1000 replicates), and branches corresponding to partitions reproduced in <50% bootstrap replicates were collapsed. The black triangle corresponds to 50 modern dog specimens.

Phylogenetic analysis demonstrated that the genomic information obtained for the Chauvet sample is high enough to show its position within the *Canis lupus* lineage (Figure 9). Furthermore, the data demonstrate robust support to the position of Chauvet among gray wolves rather than modern dogs. However, there is only weak support (bootstrap value: 50) for positioning Chauvet with Old World wolves rather than with New World wolves. We therefore performed additional analyses to address this question.

**FIGURE 10:**
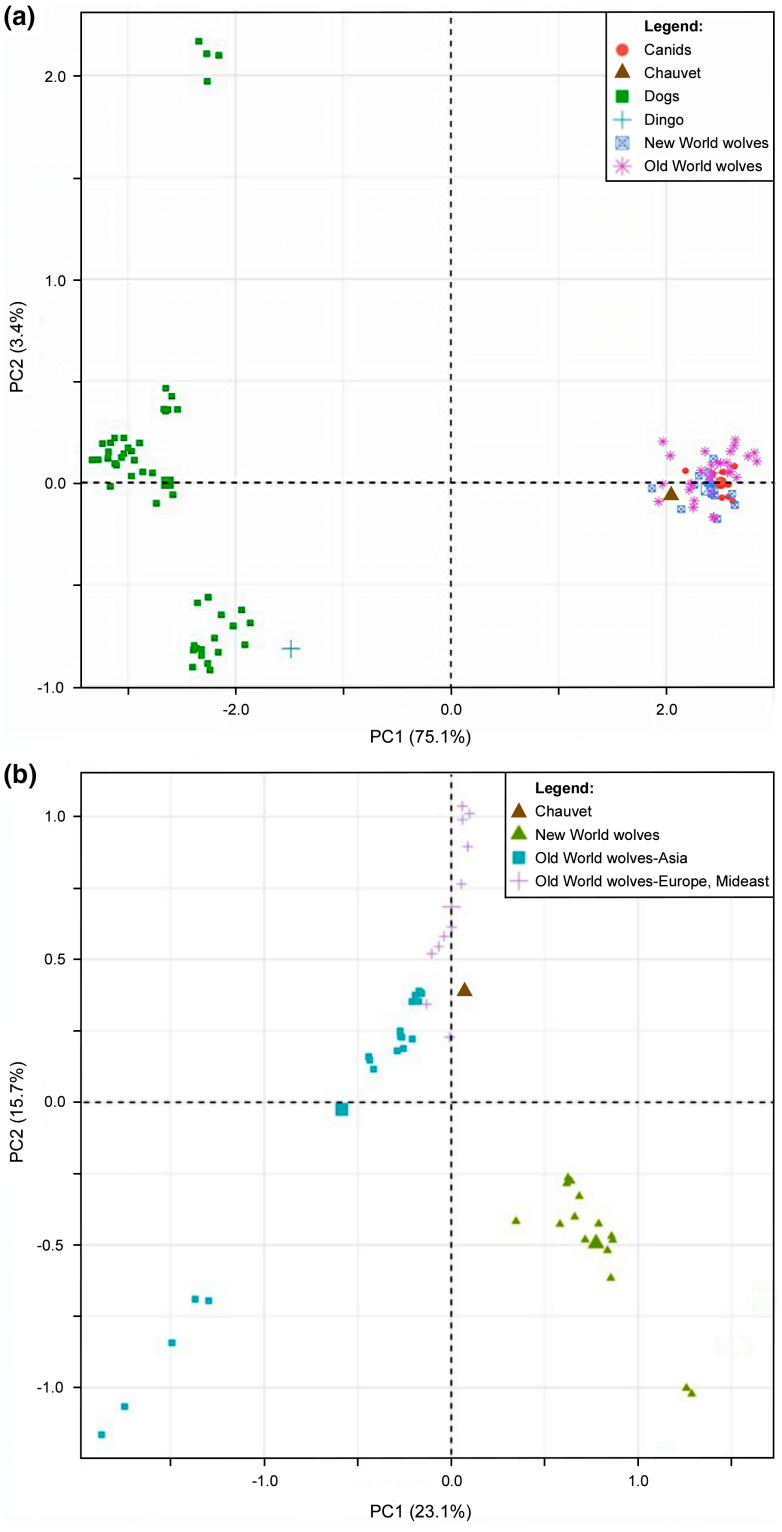
Principal component analysis (PCA) of canid nuclear SNPs. (a) PCA plot includes the Chauvet sample, modern dogs, wolves, wild canids (coyote, golden‐jackal, dhole, red fox, Andean fox), and the Dingo. (b) Plot for the analysis focused on the Chauvet sample and modern wolves.

When Principal Component Analysis (PCA) was performed using modern dogs, wolves, wild canids (coyote, golden‐jackal, dhole, red fox, Andean fox), and the Chauvet coprolite sample, dimension 1 separated domesticated dogs and the Dingo from all other canids, and dimension 2 split dogs into several subgroups but did not provide clear distinction among other canids (Figure 10a). However, when excluding dogs and wild canids, PCA efficiently separated wolves from different geographic origins (Figure 10b). New world wolves were distinctly positioned clearly apart from old world wolves; wolves from Asia and Europe‐Middle East separated with little overlap. The Chauvet coprolite was close to a subgroup of Asian and Europe‐Middle East wolves.

**FIGURE 11:**
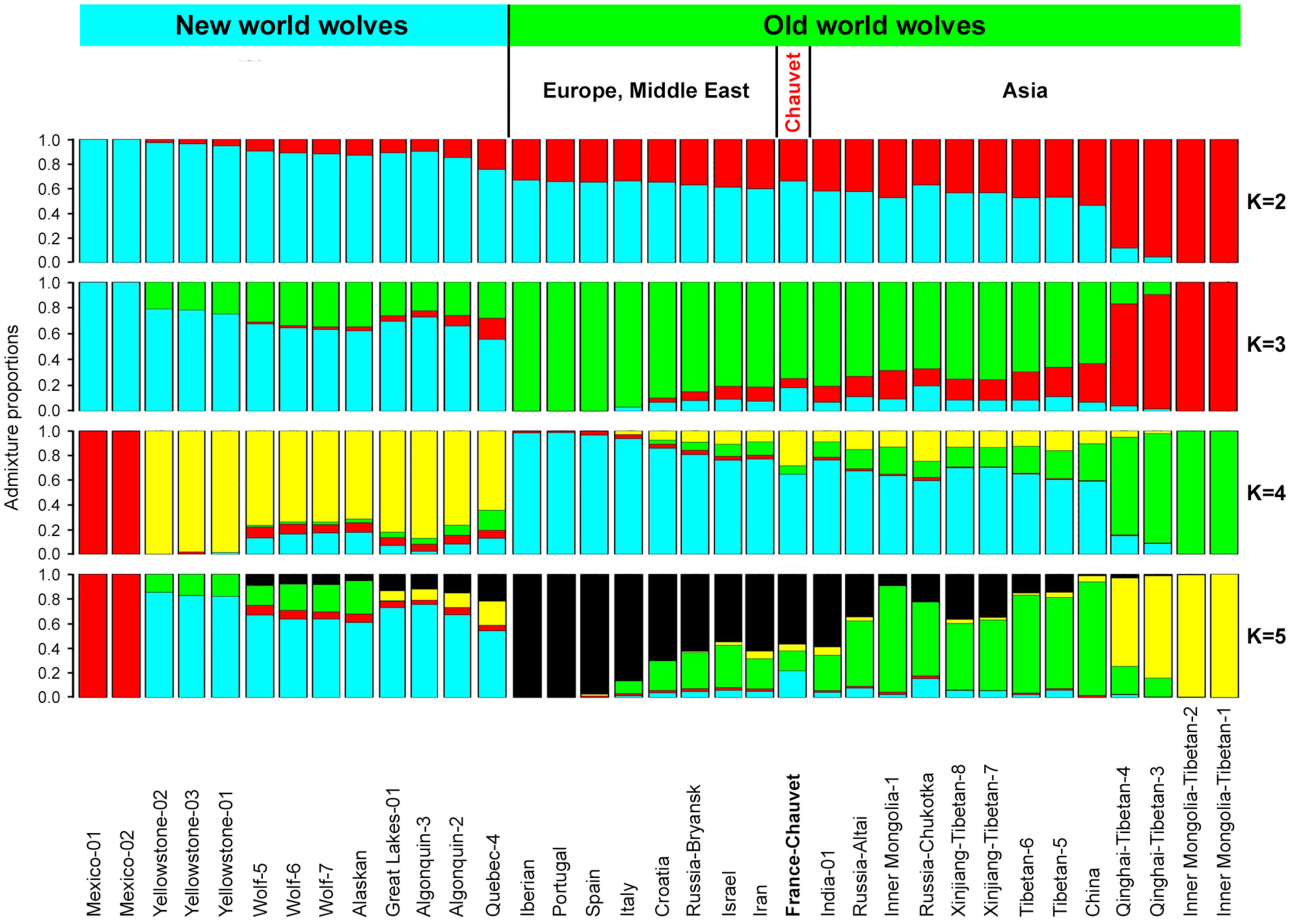
Admixture analysis of the Chauvet sample and modern wolves from around the world. The analysis was performed assuming different ancestry components, from *K* = 2 to 5, as indicated on the right part of the figure. The proportion of each color shows the estimated ancestry proportions.

We then performed admixture analysis to evaluate shared ancestry between the Chauvet canid and wolves from around the world. Old World wolves without a defined country of origin were not considered for admixture analysis. We used different numbers of estimated ancestry clusters (ranging from *K* = 2 to 5) to perform the analysis (Figure 11). In agreement with PCA data, the Chauvet specimen is more related to Old World than to New World wolves. Specifically, at the optimal *k* = 5 value defined by the Velicier's minimum average partial (MAP) test of PCangsd, the major (black) component that makes up the genome of the Chauvet sample is maximized in South‐Western European wolves (Iberian, Portugal, and Spain). The two other main components of the Chauvet genome, depicted in green and blue in Figure 11, highlight shared ancestry with a subset of Asian and North‐American wolves. By contrast, shared ancestry with Mongolian and Mexican wolves is either minimal or undetectable. A predominantly shared ancestry with specimens from Europe and the Middle East has also been reported for ancient, 14,100 to >50,000 years old Russian and Siberian specimens that correspond to extinct wolf lineages (Ramos‐Madrigal et al., 2021). In contrast to the Chauvet sample, their mitochondrial genome sequences do not belong to the divergent clade at the root of the tree (Figure 7) because they all position in the other mitochondrial clade and fall either at the base of this clade (CGG29, CGG32) or within the mitochondrial genome diversity of modern wolves (SK1, CGG33; Skoglund et al., 2015; Ramos‐Madrigal et al., 2021; Figure 7).

## 4 CONCLUSIONS

The strict conservation policies and good chemical preservation of ancient DNA in the Chauvet‐Pont d'Arc Cave have enabled us to perform the genomic analysis of a coprolite recovered in the deep cave sector, Salle Hillaire. The coprolite contained micromammal bone fragments securely dated to 36,000–34,000 cal BP, a time period corresponding to the earliest human (Aurignacian) production of artwork in Chauvet Cave and when numerous cave bears hibernated in several cave sectors in complete darkness. Shotgun DNA sequencing indicated that the most abundant animal DNA in coprolite CCH3 was canid. Reconstruction of the canid's mitochondrial genome sequence demonstrated that this individual canid belongs to a divergent clade that includes only ancient wolf specimens, notably 35,000 to 33,000‐year‐old Western European (Belgian) wolves. Analysis of nuclear genome data indicated that the Chauvet wolf specimen was a female displaying extensively shared ancestry with extant wolves from the Old World, especially European and Middle East wolves, although shared ancestry with New World wolves is also detected. Analysis of the coprolite DNA indicated that the Chauvet Cave ancient wolf's diet included the extinct cave bear, *Ursus spelaeus*, in addition to the micromammals seen physically in the coprolite. This conclusion agrees with the presence in Chauvet Cave of gnawing on long bone cave bear epiphyses (Delannoy & Geneste, 2020), a hallmark of wolf behavior, not hyena eating habits that involve crushing of bone into sharp splinters. The information obtained for the wolf's diet from DNA studies is therefore supported by archeological observation and indicates that the cave was a reservoir of food for wolves when bear carcasses were abundant, circa 35,000 years ago.

## AUTHOR CONTRIBUTIONS

**Jean‐Marc Elalouf:** Conceptualization (equal); funding acquisition (equal); investigation (equal); methodology (equal); project administration (lead); resources (equal); visualization (equal); writing – original draft (equal); writing – review and editing (equal). **Pauline Palacio:** Investigation (equal); methodology (equal). **Céline Bon:** Investigation (equal); methodology (equal). **Véronique Berthonaud:** Investigation (supporting). **Frederic Maksud:** Resources (equal). **Thomas W. Stafford:** Investigation (equal); methodology (equal); writing – original draft (equal). **Christophe Hitte:** Conceptualization (equal); data curation (lead); funding acquisition (equal); investigation (equal); methodology (equal); software (lead); visualization (equal); writing – original draft (equal); writing – review and editing (equal).

## CONFLICT OF INTEREST

The authors declare that they have no competing interests.

## Data Availability

The datasets supporting the conclusions of this article are available in the EBI and GenBank repositories. More specifically, the Illumina reads of this study have been deposited at EBI (http://www.ebi.ac.uk) under accession number ERP134157. The assembled *Canis lupus* mitochondrial genome sequence has been deposited in the GenBank database (http://www.ncbi.nlm.nih.gov/genbank) under accession number OK052506.

## Appendix

**TABLE A1:** Primers and probes used for real‐time PCR analysis of mitochondrial DNA fragments

Species	Oligonucleotide sequence	Target, size (bp)
*Canis lupus*	F, TCATCATCGCAGCTCTAGCAA R, GTTGTTGGATCCGGTTTCGT Probe, AGTACACCTCCTATTTCT	CYTB, 62
*Ursus spelaeus*	F, CCGTGCGATTAACCGCTAATA R, GTAGCTCCCCCAATTAGATGGA Probe, TACTGCAGGTCATTTAC	ATP6, 64
*Ursus arctos*	F, AAACGAGAGGTGGCAGTGGTA R, GGGCATCCGTGTAGTCATTCA Probe, AACTCACCTCAACCAAC	COX1, 62
*Martes foina*	F, CATCGCTGGATCCATAGTCCTT R, TCCCGTACCCGCCTAGTTT Probe, CAGCCGTGCTCCT	ND4, 56
*Crocuta crocuta*	F, TGGCGAGACATTATCCGAGAA R, CGCAGCCCCTTTTGTACAGT Probe, CACATTCCAGGGACACC	COX3, 68
*Coelodonta antiquatis*	F, TCTTTTCCGACCCCTTGTCA R, TGAGTGGGAGCAGTCACGTAGT Probe, CACCCTTACTGGCTCT	ND4, 60
*Cervus elaphus*	F, CATGTAGGGCGAGGCCTGTA R, AACTACTCCGATGTTTCACGTCTCT Probe, ACGGATCATATACTTTTC	CYTB, 66

*Note*: Oligonucleotides sequences are displayed in the 5′–3′ orientation. F, forward PCR primer; R, reverse PCR primer; Probe, TaqMan minor groove binder (MGB) probe labeled with 6‐carboxyfluorescein (FAM). The last column indicates the region of the mitochondrial genome amplified with each primer set and the size of the amplicon.

**TABLE A2:** Overview of DNA libraries produced for this project

Library	Amplified library	Extract (μl)	Reagents	DNA polymerase	Genoscope ID	Sequencing platform	Read length (nt)	Total reads	Mapped reads	Unique reads mapped	Uniquely mapped (% total reads)
1	1.0	CCH3T (5)	FC‐102‐1001	Phusion	AQG_BOSS	GaIIx	76	222,699,918	24,370,217	18,006,813	8.09
2	2.0	CCH3T (10)	FC‐102‐1001	Phusion	AQG_COSS	GaIIx	76	218,437,132	21,027,003	16,637,138	7.61
3	3.1 3.2	CCH3C (5)	PE‐102‐1001	Phusion Phusion U	AQG_GOSU BXW_COSU	HiSeq2000 HiSeq2500	101 101	361,259,187 1,677,388,876	18,571,200 91,090,192	17,740,817 81,113,424	4.91 4.84
4	4.0	CCH3T (10)	PE‐102‐1001	Phusion U	BXW_BOSU	HiSeq2500	101	1,261,401,653	52,638,000	50,585,075	4..01
5	5.0	CCH3T (5)	FC‐121‐4001	Phusion U	BXW_AOSW	HiSeq2500	101	570,308,101	10,760,312	10,532,020	1.85

*Note*: Column 4 provides references of Illumina reagents used to produce the library; column 5 indicates the DNA polymerase used to amplify the library; column 8 indicates number of nucleotides (nt) provided by the sequencing platform before adapters trimming; columns 9–12 corresponds to DNA reads with length ≥30 nucleotides after adapters trimming. Mapped reads correspond to reads aligned to the CanFam 3.1 dog reference genome using the criteria described in Methods.

**TABLE A3:** List of specimens used for nuclear genome analysis

Specimen	Common name, origin	Species	Specimen	Common name, origin	Species
Andean fox	Andean fox, NW	*Lycalopex culpaeus*	Wolf‐Portugal‐01	Wolf, OW	*Canis lupus*
Coyote‐1	Coyote, NW	*Canis latrans*	Wolf‐Spain‐01	Wolf, OW	*Canis lupus*
Coyote‐2	Coyote, NW	*Canis latrans*	Iberian‐Wolf	Wolf, OW	*Canis lupus*
Coyote‐3	Coyote, NW	*Canis latrans*	Wolf‐Italy‐01	Wolf, OW	*Canis lupus*
Dhole	Dhole, OW	*Cuon alpinus*	Wolf‐Croatian	Wolf, OW	*Canis lupus*
Golden jackal	Golden jackal, OW	*Canis aureus*	Wolf‐Israeli	Wolf, OW	*Canis lupus*
Red wolf‐1	Red wolf, NW	*Canis rufus*	Wolf‐Iran‐01	Wolf, OW	*Canis lupus*
Red wolf‐2	Red wolf, NW	*Canis rufus*	Wolf‐India‐01	Wolf, OW	*Canis lupus*
Afghan‐Hound‐1	Dog	*Canis lupus familiaris*	Wolf‐Altai‐Russia	Wolf, OW	*Canis lupus*
Afghan‐Hound‐2	Dog	*Canis lupus familiaris*	Wolf‐Bryansk‐Russia	Wolf, OW	*Canis lupus*
Afghan‐Hound‐3	Dog	*Canis lupus familiaris*	Wolf‐Chukotka‐Russia	Wolf, OW	*Canis lupus*
Alaskan‐Husky‐1	Dog	*Canis lupus familiaris*	Wolf‐Inner‐Mongolia	Wolf, OW	*Canis lupus*
Alaskan‐Husky‐2	Dog	*Canis lupus familiaris*	Wolf‐InnerMongolia‐Tibetan‐1	Wolf, OW	*Canis lupus*
Alaskan‐Malamute‐1	Dog	*Canis lupus familiaris*	Wolf‐InnerMongolia‐Tibetan‐2	Wolf, OW	*Canis lupus*
Alaskan‐Malamute‐2	Dog	*Canis lupus familiaris*	Wolf‐Qinghai‐Tibetan‐3	Wolf, OW	*Canis lupus*
Alaskan‐Malamute‐3	Dog	*Canis lupus familiaris*	Wolf‐Qinghai‐Tibetan‐4	Wolf, OW	*Canis lupus*
Basenji‐1	Dog	*Canis lupus familiaris*	Wolf‐Tibetan‐5	Wolf, OW	*Canis lupus*
Basenji‐2	Dog	*Canis lupus familiaris*	Wolf‐Tibetan‐6	Wolf, OW	*Canis lupus*
Basenji‐3	Dog	*Canis lupus familiaris*	Wolf‐Xinjiang‐Tibetan‐7	Wolf, OW	*Canis lupus*
Basenji‐4	Dog	*Canis lupus familiaris*	Wolf‐Xinjiang‐Tibetan‐8	Wolf, OW	*Canis lupus*
Beagle‐2	Dog	*Canis lupus familiaris*	Wolf‐Chinese	Wolf, OW	*Canis lupus*
Beagle‐3	Dog	*Canis lupus familiaris*	Grey‐Wolf‐20	Wolf, OW	*Canis lupus*
Beagle‐4	Dog	*Canis lupus familiaris*	Grey‐Wolf‐21	Wolf, OW	*Canis lupus*
Beagle‐5	Dog	*Canis lupus familiaris*	Grey‐Wolf‐22	Wolf, OW	*Canis lupus*
Border‐Collie‐10	Dog	*Canis lupus familiaris*	Grey‐Wolf‐23	Wolf, OW	*Canis lupus*
Border‐Collie‐2	Dog	*Canis lupus familiaris*	Grey‐Wolf‐24	Wolf, OW	*Canis lupus*
Border‐Collie‐7	Dog	*Canis lupus familiaris*	Grey‐Wolf‐25	Wolf, OW	*Canis lupus*
Border‐Collie‐8	Dog	*Canis lupus familiaris*	Grey‐Wolf‐26	Wolf, OW	*Canis lupus*
Chihuahua‐1	Dog	*Canis lupus familiaris*	Grey‐Wolf‐27	Wolf, OW	*Canis lupus*
Chihuahua‐2	Dog	*Canis lupus familiaris*	Wolf‐Alaskan	Wolf, NW	*Canis lupus*
Chihuahua‐3	Dog	*Canis lupus familiaris*	Wolf‐Algonquin2	Wolf, NW	*Canis lupus*
Chonggqing‐Dog	Dog	*Canis lupus familiaris*	Wolf‐Algonquin3	Wolf, NW	*Canis lupus*
Chow‐Chow‐1	Dog	*Canis lupus familiaris*	Wolf‐Great‐Lakes‐01	Wolf, NW	*Canis lupus*
Chow‐Chow‐2	Dog	*Canis lupus familiaris*	Wolf‐Mexico‐01	Wolf, NW	*Canis lupus*
Chow‐Chow‐3	Dog	*Canis lupus familiaris*	Wolf‐Mexico‐02	Wolf, NW	*Canis lupus*
Chow‐Chow‐4	Dog	*Canis lupus familiaris*	Wolf‐Unknown‐Locale	Wolf, NW	*Canis lupus*
Golden‐Retriever‐1	Dog	*Canis lupus familiaris*	Wolf‐Yellowstone‐01	Wolf, NW	*Canis lupus*
Golden‐Retriever‐2	Dog	*Canis lupus familiaris*	Wolf‐Yellowstone‐02	Wolf, NW	*Canis lupus*
Golden‐Retriever‐3	Dog	*Canis lupus familiaris*	Wolf‐Yellowstone‐03	Wolf, NW	*Canis lupus*
Golden‐Retriever‐4	Dog	*Canis lupus familiaris*	Wolf‐Quebec‐4	Wolf, NW	*Canis lupus*
Greenland‐Dog	Dog	*Canis lupus familiaris*	Wolf‐5	Wolf, NW	*Canis lupus*
Peruvian‐Inca‐Orchid	Dog	*Canis lupus familiaris*	Wolf‐6	Wolf, NW	*Canis lupus*
Saluki‐1	Dog	*Canis lupus familiaris*	Wolf‐7	Wolf, NW	*Canis lupus*
Saluki‐2	Dog	*Canis lupus familiaris*	Wolf‐8	Wolf, NW	*Canis lupus*
Saluki‐3	Dog	*Canis lupus familiaris*	Wolf‐9	Wolf, NW	*Canis lupus*
Samoyed‐1	Dog	*Canis lupus familiaris*	Wolf‐10	Wolf, NW	*Canis lupus*
Samoyed‐2	Dog	*Canis lupus familiaris*			
Shiba‐Inu‐1	Dog	*Canis lupus familiaris*			
Shiba‐Inu‐2	Dog	*Canis lupus familiaris*			
Siberian‐Husky‐1	Dog	*Canis lupus familiaris*			
Siberian‐Husky‐2	Dog	*Canis lupus familiaris*			
Siberian‐Husky‐3	Dog	*Canis lupus familiaris*			
Siberian‐Husky‐4	Dog	*Canis lupus familiaris*			
Tibetan‐Terrier‐1	Dog	*Canis lupus familiaris*			
Tibetan‐Terrier‐2	Dog	*Canis lupus familiaris*			
Xoloitzcuintli‐1	Dog	*Canis lupus familiaris*			
Xoloitzcuintli‐2	Dog	*Canis lupus familiaris*			
Dingo	Dingo	*Canis lupus dingo*			

*Note*: Genome data for the specimens are from the National Human Genome Research Institute dog genome project (Plassais et al., 2019; BioProject: PRJNA448733). NW, New World; OW, Old World.
